# Ceftriaxone, a Beta-Lactam Antibiotic, Modulates Apoptosis Pathways and Oxidative Stress in a Rat Model of Neuropathic Pain

**DOI:** 10.1155/2014/937568

**Published:** 2014-06-16

**Authors:** Bahareh Amin, Valiollah Hajhashemi, Khalil Abnous, Hossein Hosseinzadeh

**Affiliations:** ^1^Department of Pharmacology and Physiology, Faculty of Medicine, Sabzevar University of Medical Sciences, Sabzevar, Iran; ^2^Department of Pharmacology, Pharmaceutical Sciences Research Center, School of Pharmacy, Isfahan University of Medical Sciences, Isfahan, Iran; ^3^Department of Biotechnology, Pharmaceutical Research Center, School of Pharmacy, Mashhad University of Medical Sciences, Mashhad, Iran; ^4^Pharmaceutical Research Center, Pharmacodynamy and Toxicology Department, School of Pharmacy, Mashhad University of Medical Sciences, P.O. Box 1365-91775, Vakilabad Boulevard, Mashhad, Iran

## Abstract

*Purpose*. In our previous study, ceftriaxone, a beta-lactam antibiotic, elicited antinociceptive effects in the chronic constriction injury (CCI) of neuropathic pain. In this study, we assessed apoptosis and oxidative stress in the spinal cord of neuropathic rats treated with ceftriaxone. *Methods*. 45 male Wistar rats were divided as naïve, sham, normal saline-treated CCI rats, and CCI animals treated with the effective dose of ceftriaxone. Involvement of Bax, Bcl2, and caspases 3 and 9, important contributors of programmed cell death (apoptosis), was determined using western blotting at days 3 and 7. The markers of oxidative stress including malondialdehyde (MDA) and reduced glutathione (GSH) were measured on days 3 and 7. *Results*. Increased Bax/Bcl2 ratio and cleaved active forms of caspases 3 and 9 were observed in the spinal cord of CCI rats on day 3. Ceftriaxone attenuated the increased levels of Bax and cleaved forms of caspases 3 and 9, while it increased Bcl2 levels. Bax and active forms of caspases declined by day 7. Consequently, comparison among groups showed no difference at this time. CCI enhanced MDA and decreased GSH on days 3 and 7, while ceftriaxone protected against the CCI-induced oxidative stress. *Conclusion*. Our results suggest that ceftriaxone, an upregulator/activator of GLT1, could concomitantly reduce oxidative stress and apoptosis and producing its new analogs lacking antimicrobial activity may represent a novel approach for neuropathic pain treatment.

## 1. Introduction

Nerve injury induced chronic pain, often referred as neuropathic pain, is caused by a primary lesion in both central and more frequently peripheral nervous systems and is a challenging condition to treat [1]. Molecular mechanisms involved in this pathological chronic pain syndrome have currently been an area of much interest.

Excitotoxicity via elevated excitatory neurotransmitters (glutamate and aspartate) has been associated in different kinds of pain such as neuropathic pain [2]. In recent years, different studies have pointed out the role of apoptosis in neuropathic pain. In a study by de Novellis et al., apoptotic pathways were activated in rat spinal cord of sciatic nerve chronic constriction injury (CCI), and the early overexpression of proapoptotic genes and morphological changes in dorsal horn were prevented by blockade of glutamate mGlu5 receptors [3]. Afrazi et al. reported that diabetes-induced hyperalgesia in rats was attenuated by neurosteroid, allopregnanolone through inhibiting caspase-3 and decreased ratio of Bax to Bcl2 [4]. Moreover, numerous studies revealed that oxidative stress plays an important role in neuropathic pain [5, 6]. Considering that extra levels of glutamate lead to a number of deleterious effects, including excessive activation of central glutamate receptors, impairment in buffering of calcium, production of free radicals by lipid peroxidation, secondary excitotoxicity, and cell death following neuropathic pain maintaining low extracellular glutamate, is very important [7, 8]. Glutamate hemostasis is performed via high affinity transporters in which GLT 1 is the predominant subtype and accounts for clearance of the bulk of released glutamate. Downregulation of glutamate transporters is thought to be very important in various experimental neuropathic pain models [9]. Recently, ceftriaxone a third generation beta-lactam antibiotic has been discovered to have neuroprotective effects in various* in vitro* and* in vivo* studies [10, 11]. The precise primary molecular mechanism of ceftriaxone is thought to be mediated by the increased expression and activation of GLT1 in the CNS [11–13]. We and others have previously reported that ceftriaxone could attenuate pain behaviors of rats subjected to the CCI model of neuropathic pain [14, 15]. If apoptosis process and oxidative stress were linked to excitotoxicity, then treatment with ceftriaxone would be predicted to reverse spinal cord protein changes involved in the apoptosis and oxidative stress following CCI.

Therefore, the aim of the present study was to determine time course of changes in the spinal cord levels of apoptosis-related proteins. Bax, a promoting factor, Bcl2, which can prevent apoptosis, caspase-9, the initiator caspase for the activation of downstream caspases, and caspase-3, a factor in downstream of several apoptotic pathways [16], were analyzed with western blotting on days 3 and 7 after CCI. To study whether treatment with ceftriaxone was able to show antioxidant activity, we assessed spinal cord levels of MDA, the last product of lipid peroxidation [17] and glutathione, the major sulfhydryl (-SH) antioxidant, and enzyme cofactor [18], in 3 and 7 days in CCI rats.

## 2. Materials and Methods

### 2.1. Animals

Adult male Wistar strain rats weighing 220–270 g at the time of surgery were used in this experiment and randomly gathered from the animal room of the School of Pharmacy, Mashhad University of Medical Sciences, Iran. The animals were housed under standard environmental conditions (12-12 h light/dark cycle at 22°C). Rat chow and tap water were available* ad libitum*. The experimental protocol was approved by Mashhad University of Medical Sciences and performed in accordance with the Internationally Accepted Principles for Laboratory Animal Use and Care [19].

### 2.2. Drugs and Solutions

Ceftriaxone (Jaber Ebne Hayyan Pharmaceutical Co., Tehran, Iran) was dissolved in normal saline solution (0.9% NaCl) and intraperitoneally injected at the dose of 200 mg/kg. Ceftriaxone administration started when CCI was induced and continued for 7 consecutive days. Ketamine and xylazine (Alfasan Pharmaceutical Co., Woerden, Holland) were intraperitoneally injected at doses of 64 and 1.6 mg/kg, respectively.

### 2.3. CCI Surgery of Sciatic Nerve

At first, rats were anaesthetized with a cocktail of ketamine and xylazine. Mononeuropathy was induced by performing chronic constriction injury model on the left sciatic nerve of animals in accordance with the method of Bennet and Xie [20]. After the incision of the skin, the sciatic nerve was exposed and four ligatures of 4-0 gauge chromic catgut were tied loosely with an interval of 1 mm, until a slight twitching was observed in the expected hind paw. Finally, muscle and skin were separately sutured with 4-0 silk catgut and animals were placed in a warm condition until recovery. Rats in the sham group had their sciatic nerve exposed but not ligated.

### 2.4. Study Protocol

Based on our previous study, CCI led to a significant development of mechanical allodynia (4.3 ± 0.6 g versus 53 ± 6.7 g) and cold allodynia (73.3 ± 8.4% versus 8 ± 4.9%) on day 3 in comparison to sham group, as revealed by von Frey hairs and acetone drop, respectively. Pain behaviors progressively increased during the study on days 5 and 7. In that study, mechanical and cold allodynia were significantly attenuated by the dose of 200 mg/kg of ceftriaxone on days 3 and 7 [14]. Accordingly the effective antinociceptive dose of ceftriaxone (200 mg/kg) was chosen in this study.

In the present study, to examine the time course of changes in apoptosis-related proteins and oxidative stress markers (MDA and GSH) in the spinal cord of CCI rats, three animals from each group were harvested on postoperative day 3 or day 7 after the behavioral tests. Hence, 54 rats were randomly assigned into the following groups. (1, 2) The animals were subjected to CCI surgery, treated with normal saline (NS) at a dose of 1 mL/kg, and killed on day 3 or day 7 for evaluation of apoptotic factors (*n* = 3). (3, 4) In sham group, the animals underwent a similar surgery except that sciatic nerves were not ligated and treated with the normal saline and killed on day 3 or day 7 for evaluation of apoptotic factors (*n* = 3). (5, 6) CCI animals were treated with ceftriaxone (200 mg/kg, administered at a dose of 1 mL/kg) for seven days and killed on day 3 or day 7 for evaluation of apoptotic factors (*n* = 3). (7, 8) The CCI animals were treated with NS and killed on day 3 or day 7 for evaluation of MDA (*n* = 3) and GSH (*n* = 3). (9, 10) Sham-operated animals were killed on day 3 or day 7 for evaluation of MDA (*n* = 3) and GSH (*n* = 3). (11, 12) CCI animals were treated with ceftriaxone (200 mg/kg) for seven days and killed on day 3 or day 7 for evaluation of MDA (*n* = 3) and GSH (*n* = 3). (13) Naïve animals were killed for evaluation of apoptotic factors (*n* = 3), MDA (*n* = 3), and GSH (*n* = 3) contents.

For the protein extraction, the lumbar spinal cord was rapidly ejected from the vertebral column using a saline-filled syringe and then separated on dry ice. Lumbar (L4 and L5) segments were excised to measure apoptotic and oxidative stress markers considering that these segments are the major contributor to the sciatic nerve [19].

### 2.5. Western Blotting

At the time of experiment, samples were homogenized in the lysis buffer containing 50 mM Tris-HCl (pH: 7.4), 2 mM EDTA, 2 mM EGTA, 10 mM NaF, 1 mM sodium orthovanadate (Na3VO4), 10 mM *β*-glycerophosphate, 0.2% W/V sodium deoxycholate, 1 mM phenylmethylsulfonyl fluoride (PMSF), and complete protease inhibitor cocktail (Roche, Mannheim, Germany). The homogenate was sonicated on ice with three 10 sec bursts at high intensity with a 10 sec cooling period between each burst. The samples were centrifuged at 10,000 g for 10 min at 4°C. After determining protein content by Bradford assay kit and adjusting the protein content [20], each adjusted sample was mixed 1 : 1 v : v with 2x SDS blue buffer, boiled, aliquoted, and kept in the −80°C freezer.

100 micrograms of each protein extracts was separated on a 12% sodium dodecyl sulfate-polyacrylamide gel (SDS-PAGE) by electrophoresis and transferred onto polyvinylidene fluoride (PVDF) membranes. Then, blots were blocked with 5% skim milk in TBST (20 mM Tris-HCl pH 7.6, 137 mM NaCl, and 0.05% Tween-20) at 4°C overnight. Mouse monoclonal anticaspase-9 (cell signaling number 9508, 1 : 1000), rabbit polyclonal anticaspase-3 (cell signaling number 9665, 1 : 1000), rabbit polyclonal anti-Bax (cell signaling number 2772, 1 : 1000), rabbit polyclonal anti-Bcl2 (cell signaling number 2870, 1 : 1000), and rabbit polyclonal anti-*β*-actin antibodies (cell signaling number 4967, 1 : 1000) were used as a primary antibody with incubation time of about 1-2 hours at room temperature, washing three times with TBST and 1-hour incubation by rabbit horseradish peroxidase-conjugate anti-rabbit IgG (cell signaling number 7074, 1 : 2000) or anti-mouse IgG (cell signaling number 7076, 1 : 2000). Enhanced chemiluminescence (Pierce) was used to visualize the peroxidase-coated bands and Alliance 4.7 Gel Doc (UK). Densitometric analysis for protein bands was performed using UVtec software (UK). The protein levels were normalized against *β*-actin intensity.

### 2.6. Measurement of MDA Levels in the Spinal Cord of Animals

Estimation of lipid peroxidation was performed by measuring the thiobarbituric acid reactive substances as described previously [21]. At the time of experiment, each sample was weighed and homogenized in 1.15% potassium chloride solution. Then, 3 mL phosphoric acid (1%) and 1 mL TBA (0.6%) were added to 0.5 mL of homogenate in a centrifuge tube and the mixture was heated for 45 min in a boiling water bath. After cooling, 4 mL n-butanol was added to the mixture and vortex mixed for 1 min followed by centrifugation at 3000 rpm for 15 minutes. The organic layer was transferred to a fresh tube and the absorbance of pink colored product was read at 532 nm. A set of MDA standards was freshly prepared and the standard curve was constructed. The results were expressed as the nmol of malondialdehyde formed per mg of protein.

### 2.7. Measurement of GSH Levels in the Spinal Cord of Animals

Total SH groups belonging to GSH were measured using DTNB (2,2′-dinitro-5,5′-dithiodibenzoic acid) as the reagent. This reagent reacts with the SH groups to produce a yellow colored complex with a peak absorbance at 412 nm. Briefly, a 10% tissue homogenate in buffer phosphate 7.4 was mixed with an equal volume of 10% trichloroacetic acid (TCA) and vortexed. The contents were then centrifuged at 5000 rpm for 10 min. Subsequently 500 *μ*L of supernatant was mixed with a reaction mixture containing 2.5 mL 0.1 M phosphate buffer (pH 8.4) and 0.5 mL DTNB. Within 10 min, the absorbance was measured at 412 nm using a spectrophotometer. A set of GSH standards was freshly prepared using commercially available standard GSH (Sigma Chemicals, USA) and the standard curve was constructed. Levels of GSH were expressed as nmol/mg protein [22].

### 2.8. Statistics

Data were expressed as means ± SEM and statistically analyzed by one-way ANOVA followed by Tukey's* post hoc* tests, using SPSS version 13. A *P* value of < 0.05 was considered to be significant.

## 3. Results

### 3.1. Bax and Bcl2 Protein Levels

**Figure 1:**
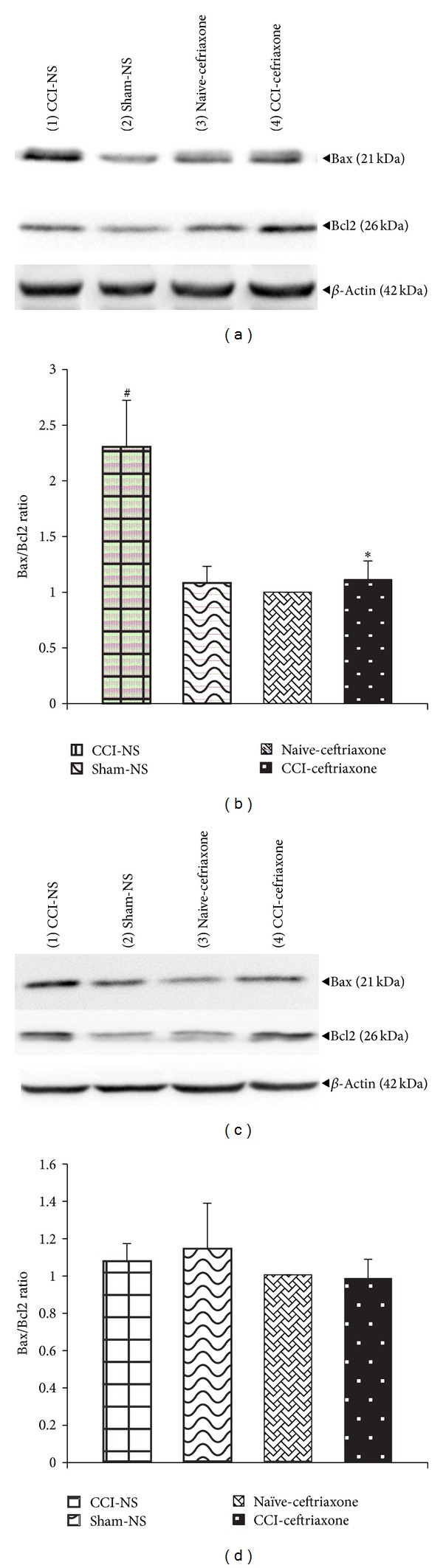
Effect of ceftriaxone on the spinal cord protein levels of Bax (21 kDa) and Bcl2 (26 kDa) and relative density of Bax/Bcl2, following western immunoblotting on days 3 (a and b, resp.) and 7 (c and d, resp.) after CCI. Administration of ceftriaxone (Cef 200 mg/kg, i.p.) started when CCI was induced and continued for 7 successive days. The semiquantitative analysis of protein levels was carried out by the “Gel Doc 2000 UV System” (Alliance 4.7). Each lane was loaded with 100 *μ*g of proteins. *β*-actin is the loading protein control. Data are mean ± SEM (*n* = 3/group). One-way ANOVA followed by Tukey's* post hoc* test was used for multiple comparisons. ^#^ *P* < 0.05 difference between CCI-NS and sham/naive groups. *P < 0.05 CCI-Cef versus NS-CCI group (control).

To analyze the amount of the apoptotic proteins, a relative protein ratio to naïve animals was used. The results of western blotting analysis indicated that lumbar spinal cord levels of the proapoptotic protein, Bax increased within 3 days following sciatic nerve CCI (control group), as compared to naïve and sham groups, while a slight but not significant decrease in the level of antiapoptotic protein, Bcl2 was observed (Figure 1(a)). Therefore, a significantly higher level of Bax/Bcl2 ratio was obtained in the spinal cord of CCI animals in comparison to naïve and sham groups (*P* < 0.05) on day 3 after surgery (Figure 1(b)). Whereas, there was no significant difference in the levels of proteins between sham and naïve animals treated with ceftriaxone. In NS-CCI animals, protein levels of Bax decreased and Bcl2 increased on day 7 after CCI. Accordingly, there was no significant difference between Bax/Bcl2 ratio of control group and that of sham and naïve groups (Figures 1(c) and 1(d)). CCI animals treated with 200 mg/kg of ceftriaxone (once daily, for 7 days) showed a significant increase in the levels of antiapoptotic protein, Bcl2, and decreased contents of Bax protein on day 3 after CCI, resulting in a significant reduction in the Bax/Bcl2 ratio as compared to control group (*P* < 0.05). No significant difference was observed in the Bax/Bcl2 ratio between ceftriaxone treated animals and that of control group on day 7 (Figure 1).

### 3.2. Caspase-9 Protein Levels

**Figure 2:**
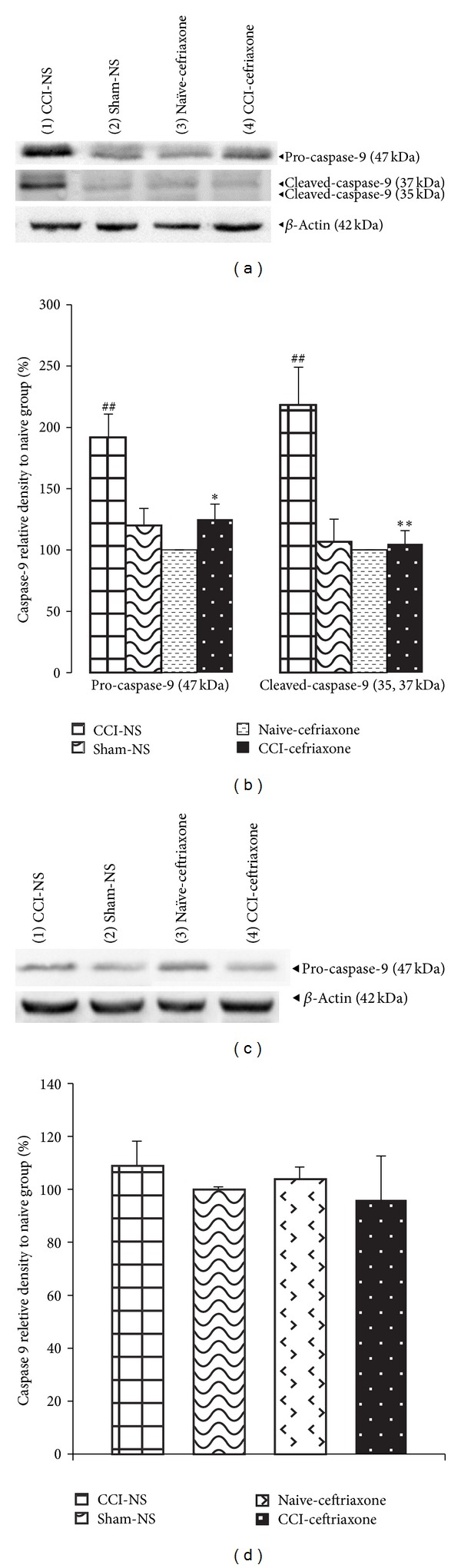
Effect of ceftriaxone on the spinal cord protein levels of procaspase-9 (47 kDa) and cleavage products (37 and 35 kDa) and relative density following western immunoblotting on days 3 (a and b, resp.) and 7 (c and d, resp.) after CCI. Administration of ceftriaxone (Cef 200 mg/kg, i.p.) started when CCI was induced and continued for 7 successive days. The semiquantitative analysis of protein levels was carried out by the “Gel Doc 2000 UV System” (Alliance 4.7). Each lane was loaded with 100 *μ*g of proteins. *β*-Actin is the loading protein control. Data were mean ± SEM (*n* = 3/group). One-way ANOVA followed by Tukey's* post hoc* test was used for multiple comparisons. ^##^ *P* < 0.01 difference between CCI-NS and sham/naive animals. *P < 0.05, **P < 0.05 CCI-Cef versus NS-CCI group (control).

Our data showed that there was no significant difference in the levels of procaspase-9 protein between sham and naïve animals treated with ceftriaxone. The expression of procaspase-9 increased on day 3 after CCI when compared to that of the sham and naïve groups (*P* < 0.01). A significant increase in the activated cleaved forms of 37 kDa and specially 35 kDa species was observed 3 days after the CCI as compared to that detected in the sham and naïve groups (*P* < 0.01) (Figures 2(a) and 2(b)). In CCI rats who received 200 mg/kg ceftriaxone (once daily, for 7 days) contents of procaspase-9 (*P* < 0.05) as well as activated forms (*P* < 0.01) decreased after 3 days of sciatic nerve injury (Figures 2(a) and 2(b)).

Levels of procaspase-9 decreased from day 3 to day 7 after surgery in normal saline CCI animals and activate cleaved forms of caspase-9 were no longer detected by day 7. As a result, no significant difference was observed among groups at this time (Figures 2(c) and 2(d)).

### 3.3. Caspase-3 Protein Levels

**Figure 3:**
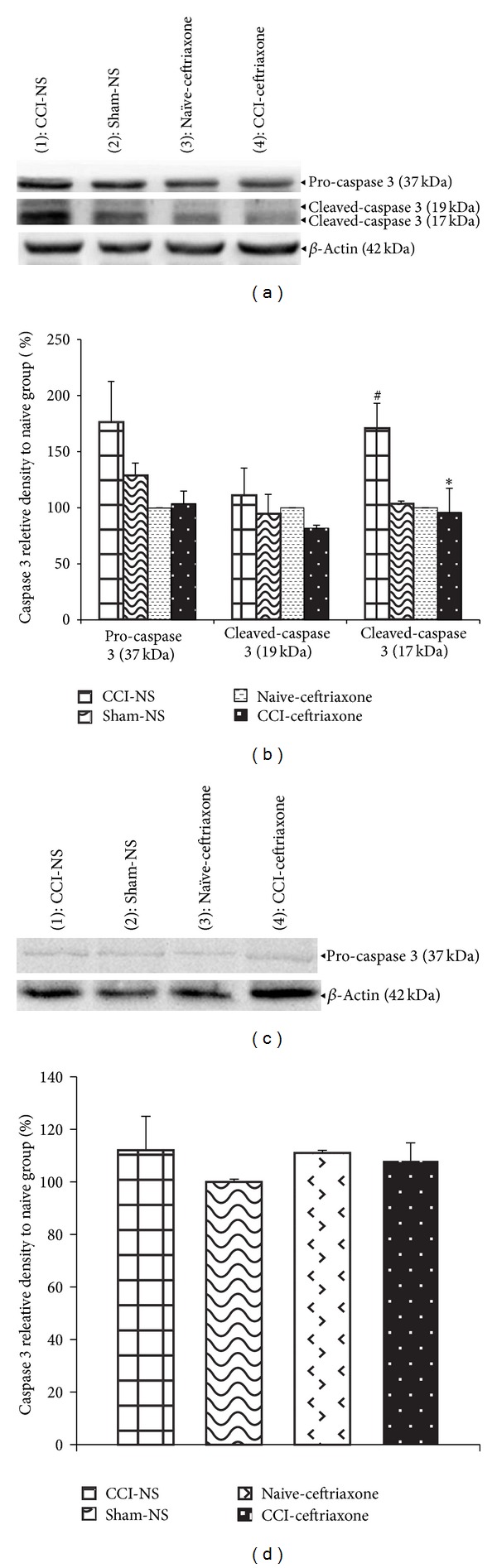
Effect of ceftriaxone on the spinal cord protein levels of procaspase-3 (37 kDa) and cleavage product (17 kDa) and relative density following western immunoblotting on days 3 (a and b, resp.) and 7 (c and d, resp.) after CCI. Administration of ceftriaxone (Cef 200 mg/kg, i.p.) started when CCI was induced and continued for 7 successive days. The semiquantitative analysis of protein levels was carried out by the “Gel Doc 2000 UV System” (Alliance 4.7). Each lane was loaded with 100 *μ*g of proteins. *β*-Actin is the loading protein control. Data were mean ± SEM (*n* = 3/group). One-way ANOVA followed by Tukey's* post hoc* test was used for multiple comparisons. ^#^ *P* < 0.05 difference between CCI-NS and sham/naive animals. *P < 0.05 CCI-Cef versus NS-CCI group (control).

On day 3 after surgery, procaspase-3 protein levels increased in the spinal cord of normal saline-treated CCI animals (control group), although not to a significant extent. Cleaved form of the protein (17 kD) was significantly increased in control group as compared to naïve and sham groups (*P* < 0.05) (Figures 3(a) and 3(b)). Despite the fact that there is no significant difference between the relative density of procaspase-3 in CCI rats and ceftriaxone treated animals, activated forms of caspase-3 were significantly attenuated by ceftriaxone (200 mg/kg), as compared to CCI NS-treated rats (*P* < 0.05) (Figures 3(a) and 3(b)). Activated cleaved forms of caspase-3 were no longer detected by day 7. Accordingly, no significant difference was observed among groups at this time (Figures 3(c) and 3(d)).

### 3.4. Malondialdehyde Levels

As indicated in Table 1, at day 3 after operation, there was a significant increase in the spinal cord MDA levels following CCI in animals that received normal saline as compared with naïve (*P* < 0.05) and sham-operated groups (*P* < 0.01). Levels of MDA remained high on day 7 after CCI. Seven days of treatment with ceftriaxone resulted in a significant reduction in the free radical-mediated lipid peroxidation on days 3 and 7 as indicated by the decreased levels of MDA compared to CCI saline-treated group (*P* < 0.05).

### 3.5. Glutathione Levels

After 7 days of CCI, GSH levels were significantly decreased in the spinal cord of animals receiving normal saline, in relation to that of the sham and naïve groups (*P* < 0.01). Ceftriaxone significantly increased antioxidant power by a significant increase in the levels of GSH on day 7 in the spinal cord of CCI animals (*P* < 0.05) (Table 1).

## 4. Discussion

Antinociceptive effects of ceftriaxone have been shown in our previous study [14]. GLT1 upregulation and activation are assumed to be the main mechanisms of neuroprotective effects of ceftriaxone in various experimental models including neuropathic pain [7, 11, 12, 15]. In the present study, the time course alterations in apoptosis markers (Bax : Bcl2), as well as caspases 3 and 9, were evaluated on days 3 and 7 after CCI. We also measured oxidative markers, MDA and GSH, in the lumbar spinal cord of animals on the 3rd and 7th days after CCI. On day 3, after operation, CCI induced elevated level of Bax protein, while a slight decrease was observed in the Bcl2 protein level. Therefore, a significant elevation was detected in the Bax/Bcl2 ratio in animals subjected to CCI at this time. Bax is representative apoptotic protein in the Bcl2 family and responsible for the subsequent activation of caspases and cell apoptosis. Indeed, the ratio of Bax to Bcl2 can determine the susceptibility of cells to cell death. A functional imbalance between proapoptotic Bax and antiapoptotic Bcl2 has been implicated following chronic constriction of sciatic nerve in rats [23]. As there was not a significant difference between apoptotic protein levels of naïve animals treated with ceftriaxone relative to those of sham-operated animals, it could be suggested that ceftriaxone has no direct effect on apoptotic proteins. However, ceftriaxone reduced Bax and increased Bcl2 levels in CCI animals and as a result a significant decrease was detected in the Bax/Bcl2 ratio of spinal cord in the sciatic nerve CCI rats on postoperative day 3. In this study, we found a significant induction of cleaved forms of caspases 3 and 9 on day 3 after CCI followed by a decrease at day 7. In agreement with this, a recent study by Wu et al. showed that treatment of CCI animals with inhibitor of caspase-3 and siRNA targeting caspase-3 significantly inhibited the apoptosis of neurons and the thermal hyperalgesia following sciatic nerve ligation [24]. Joseph and Levine showed that caspase signaling pathway contributed to the pain induced in two models of painful peripheral neuropathy [16]. In CCI animals treated with normal saline, Bax/Bcl2 ratio of spinal cord declined thereafter on day 7. There were also no detectable cleaved forms of caspases 3 and 9 in the spinal cord of NS-CCI animals. Thereafter, there was no difference among CCI-vehicle, sham, naïve, and CCI-ceftriaxone treated groups by day 7. Our data in the present study supports the evidence that, in the CCI of sciatic nerve, the development of neuropathic pain may be associated with the activation of apoptosis process [16, 24]. However, depending on the time point of study, there are differences in pattern of apoptosis occurrence. As the antiapoptotic protein, Bcl2, increased in the control group, it seems that as a modulatory mechanism apoptotic process via mitochondria is limited to the first few days after nerve injury which is consistent with some studies. An early apoptosis (2-3 days after CCI) occurred transiently by the increased ratio of Bax/Bcl2 genes in a study by de Novellis and collogues. An inversed pattern of Bcl2 family genes expression was detected at later stages [3]. Thus, there was a significant lowering in Bax/Bcl2 and Bcl-Xs/Bcl-xL ratios over time as a consequence of increased expression of antiapoptotic Bcl2 and Bcl-xL. In a study by Costa et al., increase in the ratio between pro- and antiapoptotic gene Bax/Bcl2 expression in the spinal cord of neuropathic rats was also limited to the first few days following nerve injury [25]. Siniscalco and coworkers revealed that the levels of Bax, apoptotic protease-activating factor-1 (apaf-1), nestin, GFAP, and caspase-7 mRNA increased in the dorsal horn spinal cord by 3 days after CCI. At 7 days after CCI, only overexpression of Bcl2, nestin, and GFAP mRNA was observed [26]. In a study by Rezende et al., expression of Bax gene and Bax protein increased, while the expression of Bcl2 RNA was decreased in 5 days sciatic nerve transection rats [27].

Despite the decreased levels of proapoptotic proteins after 7 days of CCI, elevated levels of MDA and decreased levels of GSH remained in the spinal cord of 7-day CCI rats, which was consistent with the development of behavioral data in our previous study. Consequently, it seems that other mechanisms other than apoptosis through mitochondrial pathways at this time contribute to the behavioral parameters of neuropathic pain. Moreover, the important contributory role of oxidative stress is supported in the pathogenesis of neuropathic pain [6, 28]. However, it might be possible that apoptosis process again is activated after the time course of our study. Hence, taking more stage samples such as days 10 or 14 may help to better characterize time course of apoptotic factors activation. Treatment with ceftriaxone in CCI rats decreased spinal cord levels of MDA and increased contents of GSH, on days 3 and 7 after CCI, as compared with the vehicle treated control group. It has been shown that increased levels of glutamate with the prevention of cysteine uptake into the cells lead to cysteine and glutathione depletion from cells which is subsequently accompanied by an increase in reactive oxygen species (ROS). On the other hand, depletion of GSH is correlated with glutamate excitotoxicity [29]. Glutamate transporters contribute not only to the prevention of toxicity caused by glutamate but also to the stimulation of cysteine uptake through the cysteine/glutamate antiporter and also generation of GSH by providing intracellular glutamate [30].

Although the exact mechanism of antioxidant and antiapoptotic effects of ceftriaxone was not evaluated in our study, it seems that ceftriaxone through different mechanisms and pathways shows neuroprotective effects. For example, in a study by Sari et al., ceftriaxone reduced ethanol consumption in an animal model of alcohol abuse; however, only the highest doses increased the GLT1 expression. They suggested that ceftriaxone may have additional pharmacological effects independent of the activation and/or upregulation of GLT1 [31]. An* in vitro* study showed that ceftriaxone treatment increased GSH and glutamate/cystine-antiporter levels and suggested that neuroprotective effects of ceftriaxone might relate more strongly to activation of the antioxidant defense system [32]. Chu et al. revealed that ceftriaxone protected against focal cerebral ischemia by increasing the levels of GLT1 as well as reducing the levels of proinflammatory cytokines (tumor necrosis factor), matrix metalloproteinase 9 (MMP-9), and activated caspase-9 [33]. In a study by Ramos and coworkers, ceftriaxone inhibited astrocyte activation by downregulation of GFAP as well as upregulation of GLT1 expression, in CCI animals [34]. MacAluso et al. reported that a single dose of ceftriaxone but not cefazolin, a structurally similar cephalosporin antibiotic to ceftriaxone, produced analgesia in patients with painful neuropathies and mouse models of inflammatory or postsurgical pain [35]. It has previously been demonstrated that increased expression of GLT1 by ceftriaxone occurs after a few days [8, 11].

## 5. Conclusion

Taken together, ceftriaxone as a GLT1 upregulator/activator could concomitantly prevent lipid peroxidation and oxidative stress-mediated apoptosis and activate the antioxidant defense system with restoring the levels of GSH. Ceftriaxone and new drugs that act in a similar manner but without antibiotic properties could be a suitable approach for ameliorating and potentially preventing a wide range of neurodegenerative diseases caused by glutamate excitotoxicity.

## Figures and Tables

**Table 1:** The effect of intraperitoneal ceftriaxone (200 mg/kg) on the levels of malondialdehyde (MDA) and glutathione (GSH) in the lumbar spinal cord of 3- and 7-day CCI rats. Animals were treated with ceftriaxone for 7 days and administration started when CCI was induced. Data are presented as mean ± SEM (*n* = 3). One-way ANOVA followed by Tukey's *post hoc* test was used for multiple comparisons. ^##^ *P* < 0.01, ^#^ *P* < 0.05 difference between CCI-NS and sham/naive animals. ∗*P* < 0.05 CCI-Cef group versus NS-CCI group (control).

	Control group (normal saline CCI rats)	Sham group	Naive group (intact rats with daily 200 mg/kg ceftriaxone)	Treated group (CCI rats with daily 200 mg/kg ceftriaxone)
MDA (nmol/mg protein)				
Day 3	12.9 ± 2.5^##^	4.3 ± 0.23	4.7 ± 0.23	5.9 ± 1.2∗
Day 7	10.7 ± 2.1^#^	3 ± 0.16	3.1 ± 0.17	4.5 ± 1.2∗
GSH (nmol/mg protein)				
Day 3	37.7 ± 10.4	49 ± 11	41 ± 3.4	42.5 ± 3.8
Day 7	33.5 ± 4.6^##^	55.2 ± 2.5	48.9 ± 2.3	40.6 ± 2.4∗
